# Unsupervised Segmentation of Bolus and Residue in Videofluoroscopy Swallowing Studies

**DOI:** 10.3390/jimaging11100368

**Published:** 2025-10-17

**Authors:** Farnaz Khodami, Mehdy Dousty, James L. Coyle, Ervin Sejdić

**Affiliations:** 1 Faculty of Electrical and Computer Engineering, University of Toronto, 10 King’s College Rd, Toronto, ON M5S 1A1, Canada; mehdy.dousty@mail.utoronto.ca (M.D.); ervin.sejdic@utoronto.ca (E.S.); 2 Vector Institute, 108 College St, Toronto, ON M5G 0C6, Canada; 3 School of Health and Rehabilitation Sciences, University of Pittsburgh, Forbes Tower, 3600 Atwood St, Pittsburgh, PA 15260, USA; jcoyle@pitt.edu; 4 North York General Hospital, 4001 Leslie St, North York, ON M2K 1E1, Canada

**Keywords:** autoencoder, bolus tracking, convolutional neural network, dysphagia, machine learning, positional encoding, residue detection, unsupervised learning, unsupervised segmentation, videofluoroscopy swallowing study

## Abstract

Bolus tracking is a critical component of swallowing analysis, as the speed, course, and integrity of bolus movement from the mouth to the stomach, along with the presence of residue, serve as key indicators of potential abnormalities. Existing machine learning approaches for videofluoroscopic swallowing study (VFSS) analysis heavily rely on annotated data and often struggle to detect residue, which is visually subtle and underrepresented. This study proposes an unsupervised architecture to segment both bolus and residue, marking the first successful machine learning-based residue segmentation in swallowing analysis with quantitative evaluation. We introduce an unsupervised convolutional autoencoder that segments bolus and residue without requiring pixel-level annotations. To address the locality bias inherent in convolutional architectures, we incorporate positional encoding into the input representation, enabling the model to capture global spatial context. The proposed model was validated on a diverse set of VFSS images annotated by certified raters. Our method achieves an intersection over union (IoU) of 61% for bolus segmentation—comparable to state-of-the-art supervised methods—and 52% for residue detection. Despite not using pixel-wise labels for training, our model significantly outperforms top-performing supervised baselines in residue detection, as confirmed by statistical testing. These findings suggest that learning from negative space provides a robust and generalizable pathway for detecting clinically significant but sparsely represented features like residue.

## 1. Introduction

Swallowing is a vital yet complex physiological function essential for safe nutrition and airway protection. It requires the tightly coordinated action of numerous muscles and neural pathways. Swallowing difficulties—collectively termed dysphagia—can arise from a range of causes, including age-related functional decline [1], neurological disorders [2], head and neck cancers and their treatments [3], structural abnormalities, and iatrogenic complications following surgery. If left untreated, dysphagia can lead to serious consequences such as aspiration pneumonia, malnutrition, dehydration, and diminished quality of life [4]. As such, early detection and objective assessment of swallowing impairments are critical for guiding appropriate clinical intervention.

When patients present with symptoms suggestive of dysphagia, they are typically referred to a speech-language pathologist for clinical evaluation. This usually begins with a case history and sensorimotor examination, followed by instrumental diagnostic tests. Several objective modalities are employed in clinical settings, including fiber-optic endoscopic evaluation of swallowing [5], manometry [6], and surface electromyography [7]. However, the videofluoroscopic swallowing study (VFSS) remains the most widely adopted gold standard [8]. In VFSS, patients swallow various barium-labeled boluses while X-ray imaging captures a dynamic video of the swallowing process. The resulting fluoroscopic frames enable clinicians to assess the movement and coordination of anatomical structures involved in swallowing and to identify potential abnormalities.

**Figure 1:**
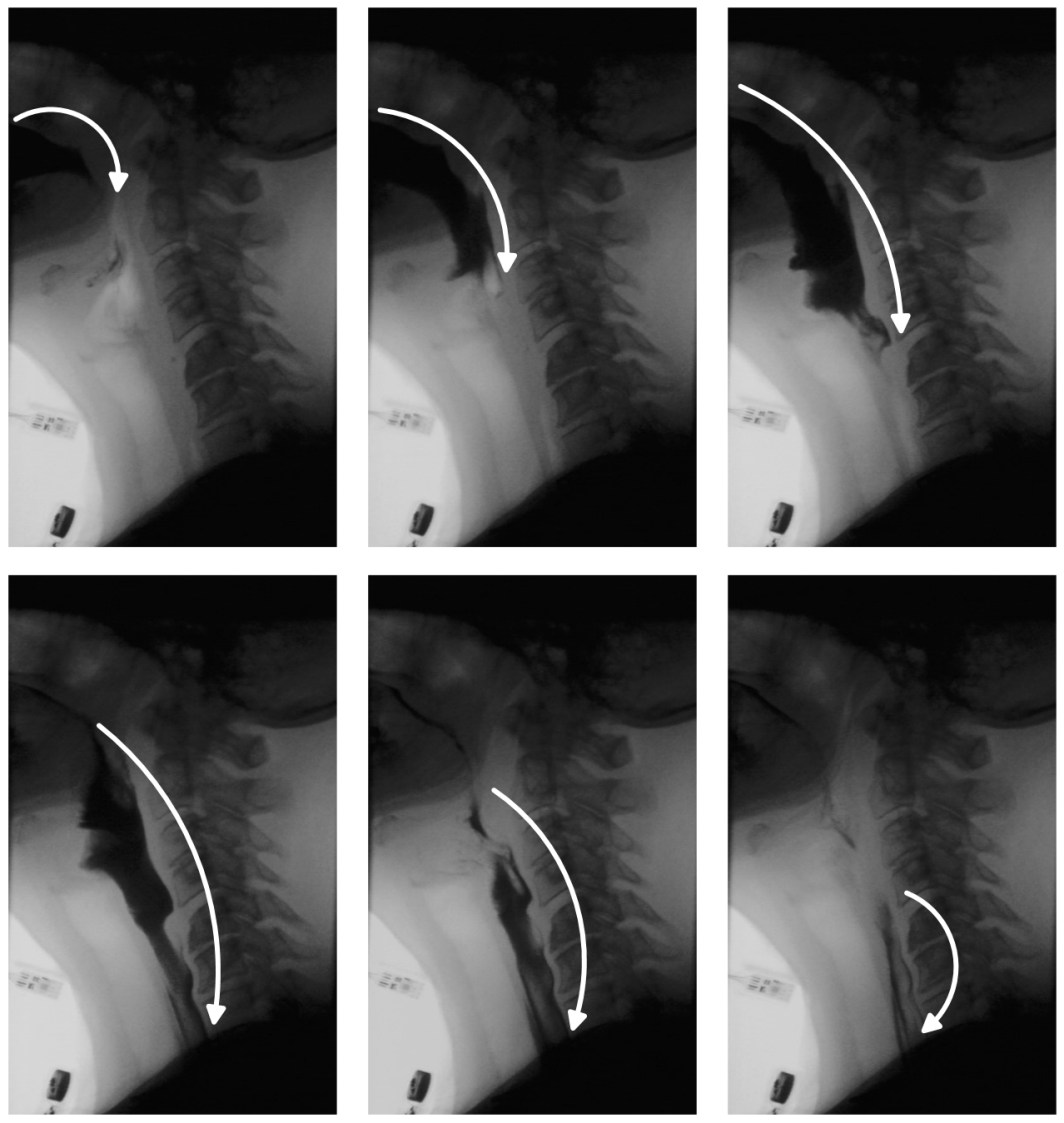
Representative VFSS frames illustrating the progression of the bolus through the oral, pharyngeal, and esophageal phases of swallowing.

One of the critical aspects of VFSS analysis is bolus tracking, which enables clinicians to assess the safety and efficiency of swallowing [9]. In a healthy swallow, the bolus moves smoothly and continuously from the oral cavity to the esophagus and into the stomach (Figure 1). In individuals with swallowing disorders, however, this movement may deviate from the normal trajectory, leading to complications. The bolus may be misdirected into the nasal cavity, fragment into smaller portions swallowed sequentially, or enter the pharynx while the airway is unprotected—raising the risk of aspiration pneumonia. In other cases, part of the bolus may remain in the oral or pharyngeal region, resulting in residue that can cause postprandial choking. While residue detection is as clinically important as bolus tracking, it has received comparatively less attention in research due to the inherent challenges in identifying small, low-contrast regions within complex anatomical backgrounds. Traditionally, clinicians manually review VFSS recordings, examining key frames to track bolus movement and identify residue. This process is time-consuming, susceptible to observer fatigue and variability, and requires specialized expertise, limiting scalability. Automated bolus tracking systems offer a promising alternative, providing more standardized and objective assessments. In addition to reducing manual workload, they facilitate quantification of important swallowing parameters, such as bolus transit time, penetration, and aspiration events.

In this study, we propose a machine learning-based approach for bolus segmentation and residue detection using a convolutional autoencoder trained exclusively on videofluoroscopy frames that do not contain bolus material. This design allows the model to learn the underlying anatomical background, enabling deviations—such as the presence of bolus—to be detected as anomalies during inference. By employing a customized loss function and incorporating a sufficient number of no-bolus frames during training, the model learns to differentiate bolus presence from other physiological movements occurring during swallowing, such as head motion or hyoid bone displacement. Consequently, the network selectively fails to reconstruct bolus regions during testing, while preserving reconstruction fidelity for normal anatomical features—an effect further reinforced through targeted postprocessing. Importantly, this approach requires only unlabeled no-bolus frames from diverse participants, promoting robust generalization across unseen subjects and eliminating the need for pixel-level annotations required by supervised methods.

To enhance segmentation quality, our model employs positional encoding as a strategy to capture global contextual information, addressing a fundamental limitation of convolutional neural networks, which primarily focus on local features [10]. While transformer-based architectures are capable of capturing such global dependencies, they often demand extensive training data and incur high computational costs. By contrast, our approach leverages fixed sinusoidal encodings that inject spatial location into the input representation without requiring additional learnable parameters. Each pixel is augmented with its corresponding encoding, allowing the model to distinguish between visually similar features appearing in different parts of the image. This spatial sensitivity is especially beneficial in anomaly detection, where deviations from expected visual patterns are not solely based on appearance but also on spatial context. Comparative results demonstrate that the use of positional encoding improves spatial awareness in the reconstruction process, leading to fewer false positives in bolus segmentation.

To evaluate model performance, we obtained a set of manually annotated images from speech-language pathologists indicating the locations of the bolus and bolus residue. The model’s output was quantitatively assessed using Intersection over Union (IoU) and Dice Similarity Coefficient (DSC), and benchmarked against both a fully supervised baseline and an identical unsupervised approach lacking global context integration. For bolus segmentation, our model achieves an IoU of 61% and a DSC of 72%, highlighting its ability to perform effectively with minimal annotation requirements. To ensure practical feasibility, we also report computational metrics, including inference speed and parameter count.

Beyond bolus localization, our model is also effective in detecting bolus residue. This is one of the first features assessed by speech-language pathologists during VFSS interpretation, as it may indicate impaired bolus propulsion, reduced pharyngeal contraction, or compromised airway protection mechanisms. To the best of our knowledge, no prior study in this area has reported quantitative results for automated residue segmentation on VFSS data. Residue regions are typically smaller in size and exhibit lower contrast relative to surrounding anatomy, making them difficult to detect. Despite these challenges, our model achieves an IoU of 52% and a DSC of 66% for residue segmentation, demonstrating its potential to fill this critical gap and support more efficient VFSS interpretation.

The main contributions of this work are as follows:

- We introduce an unsupervised autoencoder-based framework for bolus segmentation and residue detection in VFSS, removing the need for pixel-level annotations.
- We enhance reconstruction with positional encoding, which improves global contextual awareness and reduces false positives.
- We provide the first quantitative evaluations of automated residue segmentation in VFSS, addressing a clinically important but underexplored task.
- We benchmark against supervised and unsupervised baselines while also reporting computational efficiency, demonstrating both accuracy and practicality for clinical use.

## 2. Related Work

The application of machine learning to medical imaging has significantly advanced automated analysis by improving accuracy, efficiency, and reproducibility [11,12]. In the context of swallowing assessment, several works have explored VFSS analysis for bolus tracking and segmentation.

Caliskan et al. [13] employed Mask R-CNN with a pretrained ResNet101 backbone, achieving a mean average precision of 0.49 and an IoU of 0.71 for bolus segmentation. Similarly, Li et al. [14] evaluated U-Net [15] and U-Net++ [16] architectures, incorporating diverse encoder–decoder backbones such as MobileNetV2 [17], VGGNet [18], ResNet [19], DenseNet [20], Inception-ResNet [21], and EfficientNet [22]. These models were benchmarked on segmentation accuracy, computational complexity, and inference time. While these supervised methods achieved promising performance, they require extensive pixel-level annotations, which are labor-intensive and prone to inter-rater variability. Annotating segmentation masks is particularly challenging in VFSS due to low-contrast regions and ambiguous anatomical boundaries [23].

To reduce annotation demands, Bandini et al. [24] proposed a weakly supervised method for localizing the bolus during the pharyngeal phase using Grad-CAM with multiple pretrained backbones, including VGG16, InceptionV3, and ResNet50. The resulting activation maps were refined using thresholding and morphological operations. Although effective, this method still depends on labeled data and does not address bolus residue detection, which remains comparatively underexplored.

Beyond swallowing-specific applications, recent advances in image segmentation have been driven by transformer- and diffusion-based architectures. Transformer-based medical object detection has become one of the hot spots in this field [25]; however, we did not find studies that applied transformers in an unsupervised way for segmentation. Diffusion models have also gained significant popularity, with several works exploring their application to medical image segmentation and reporting promising results [26,27,28]. The main limitation of diffusion-based approaches is their requirement for very large training datasets, which poses a challenge in medical domains such as swallowing assessment where publicly available datasets are scarce and access to sufficiently large collections is difficult.

## 3. Materials and Methods

### 3.1. Dataset

The dataset used in this study comprises videofluoroscopic swallowing sequences collected from human participants, including both healthy individuals and those with swallowing difficulties. Each subject was instructed to swallow a specified bolus in a single attempt; however, some performed two to three swallows within the same recording, separated by short intervals. No postprocessing (e.g., denoising or frame interpolation) was applied. Instead, each sequence was decomposed into individual frames to enable frame-level analysis.

For model training, 10,000 bolus-free frames were extracted from 37 participants (mean age: 62.8 ± 10.8 years; 15 males, 22 females). To evaluate generalizability, an independent set of 305 frames from ten previously unseen participants (mean age: 31.6 ± 7.1 years; 4 males, 6 females) was used for validation and testing. This subject-wise split was designed to assess the model’s performance across anatomically diverse cases and reduce the risk of overfitting. The test set included both bolus-present and bolus-absent frames to ensure the model detects bolus regions accurately while avoiding false-positive predictions in bolus-free frames. The study protocol was approved by the University of Pittsburgh Institutional Review Board (IRB #12080498), and written informed consent was obtained from all participants.

### 3.2. Methodology

**Figure 2:**
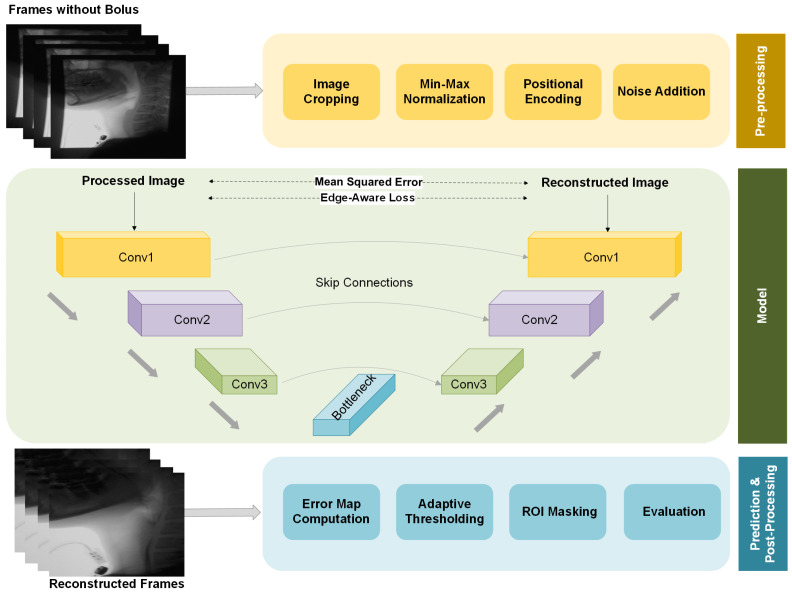
Architecture of the proposed unsupervised framework for bolus and residue segmentation. The model is trained to reconstruct bolus-free frames, allowing regions containing bolus or residue to be isolated as reconstruction anomalies during inference. This facilitates robust localization even in anatomically diverse cases.

The overall structure of our proposed framework, including preprocessing, model architecture, and postprocessing, is illustrated in Figure 2. We provide a more detailed explanation of each component in the following sections.

### 3.3. Preprocessing

Prior to training, the images undergo several preprocessing steps. First, black boundary regions are cropped to remove irrelevant areas and ensure that the model focuses on anatomical structures. This step also helps eliminate any private information, such as participant names or identifying details that might be present in the frame borders, addressing privacy concerns. Additionally, it prevents the model from learning unintended priors from extraneous information. The images are then resized to 256 × 256 pixels for consistency across the dataset. To minimize variations caused by differences in imaging conditions, min-max normalization is applied, scaling pixel intensities to a consistent range across frames. This helps stabilize brightness and contrast, enhancing the model’s robustness to variations in imaging quality.

A key enhancement in our preprocessing strategy is the integration of positional encoding, which provides global spatial context to the model. While our approach is built upon a CNN-based autoencoder, standard CNN architectures primarily focus on local features, limiting their ability to distinguish between spatially similar but contextually different regions. During preliminary experiments without positional encoding, we observed that the model was partially able to reconstruct the bolus, even though bolus-containing frames were not used during training. This unintended reconstruction likely stemmed from similarities in color, texture, or other visual features between the bolus and certain anatomical regions in the training set. As a result, the model did not consistently treat the bolus as an anomaly during inference. To address this, we incorporated positional encoding into the input representation, allowing the model to learn location-dependent feature distributions. This addition enhances spatial awareness, helping the model more accurately identify the bolus as a spatially inconsistent structure and reducing false negatives in segmentation.

**Figure 3:**
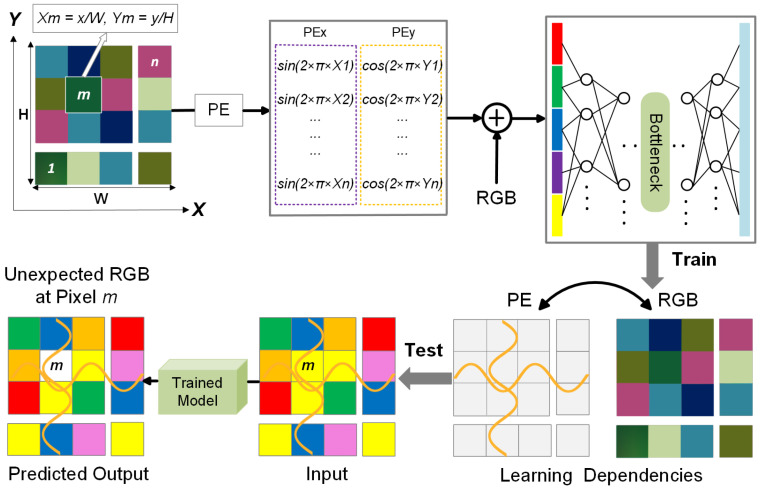
Illustration of the positional encoding process. Sinusoidal functions are applied over spatial coordinates, and the resulting positional encoding channels are concatenated with the input image. For generalizability, this process is demonstrated in the context of RGB data.

Positional encoding is computed using sinusoidal and cosine functions over the spatial dimensions:

(1) $$PE\left(x,y\right)=\left[\sin\left(2\pi \frac{x}{W}\right),\cos\left(2\pi \frac{y}{H}\right)\right]$$

where *H* and *W* denote the image height and width, respectively. The resulting encoding values are concatenated as additional input channels alongside the grayscale fluoroscopic image, enabling the model to better capture spatial relationships and location-dependent patterns. A schematic visualization is provided in Figure 3, illustrating how positional encoding assists in identifying anomalies.

When training an autoencoder for reconstruction-based anomaly detection on VFSS frames, the input to the encoder is one of the following:

- RGB only: $x\left(x,y\right)\in R^{3}$;
- RGB + positional encoding (PE): $v\left(x,y\right)\in R^{5}$, where

v(x,y)=[R(x,y),G(x,y),B(x,y),sin(2πx/W),cos(2πy/H)]

We compare two models:

- $f_{\mathrm{noPE}}:R^{3}\to R^{3}$
- $f_{\mathrm{PE}}:R^{5}\to R^{3}$

Each model is trained to minimize the reconstruction loss over the dataset D, defined as follows:

(2) $$\min_{\theta }\;E_{(x,y)∼D}{∥f_{\theta }\left(\cdot \right)-I\left(x,y\right)∥}^{2}$$

where θ denotes the trainable parameters of the model, and I(x,y) represents the original RGB intensity at pixel location (x,y).

We model the true RGB distribution at a given pixel location $\left(x_{0},y_{0}\right)$ as a random variable:

(3) $$I\left(x_{0},y_{0}\right)∼P_{x_{0},y_{0}}=N\left(\mu_{x_{0},y_{0}},\Sigma_{x_{0},y_{0}}\right)$$

Here, $\mu_{x_{0},y_{0}}\in R^{3}$ denotes the expected RGB value at location $\left(x_{0},y_{0}\right)$, and $\Sigma_{x_{0},y_{0}}\in R^{3\times 3}$ represents the covariance of RGB values observed across the training dataset at that location. These parameters characterize the spatially localized appearance statistics of the background anatomy.

We consider two autoencoder models, each parameterized by θ, and trained to minimize the reconstruction loss:

- Model A (No PE): $f_{\mathrm{noPE}}\left(\cdot ;\theta \right):R^{3}\to R^{3}$, which learns a *global distribution*:
  (4) $$f_{\mathrm{noPE}}\left(x;\theta \right)\approx \mu_{\mathrm{global}}=E_{\left(x,y\right)}\left[\mu_{x,y}\right]$$
  where $\mu_{\mathrm{global}}\in R^{3}$ is the global mean RGB vector computed by averaging across all spatial locations in the training set. Since no spatial information is provided, the model attempts to reconstruct based solely on global appearance statistics.
- Model B (With PE): $f_{\mathrm{PE}}\left(\cdot ;\theta \right):R^{5}\to R^{3}$, which learns a *location-conditioned distribution*:
  (5) $$f_{\mathrm{PE}}\left(\left[x,\mathrm{PE}\left(x,y\right)\right];\theta \right)\approx \mu_{x,y}$$
  where $\mu_{x,y}$ is the expected RGB value at position (x,y), estimated from the training data. By incorporating positional encoding, this model can learn spatially dependent RGB priors, enabling it to distinguish between anatomically similar features appearing in different locations.

At inference time, we assume a test frame contains an anomaly—such as bolus material—at a specific spatial location $\left(x_{0},y_{0}\right)$, where such a pattern was never observed during training. We model this as follows:

(6) $$I^{\prime }\left(x_{0},y_{0}\right)=\mu_{x_{0},y_{0}}+\Delta ,\;\mathrm{with}\;∥\Delta ∥\gg 0$$

Here, $\Delta \in R^{3}$ represents a significant deviation from the learned mean appearance at that location, indicating a potential anomaly.

For clarity, we express the reconstruction error using the mean squared error (MSE) formulation, though the underlying reasoning generalizes to other reconstruction losses, including the hybrid objective used in practice.

We evaluate how each model responds to this perturbation:

- With PE:
  (7) $$\begin{array}{cc} {\mathrm{Error}}_{\mathrm{PE}} & ={∥f_{\mathrm{PE}}\left(\left[I^{\prime }\left(x_{0},y_{0}\right),\mathrm{PE}\left(x_{0},y_{0}\right)\right];\theta \right)-I^{\prime }\left(x_{0},y_{0}\right)∥}^{2}\\ \; & ={∥\mu_{x_{0},y_{0}}-\left(\mu_{x_{0},y_{0}}+\Delta \right)∥}^{2}={∥\Delta ∥}^{2} \end{array}$$
  The model expects $\mu_{x_{0},y_{0}}$ at this specific location, and reconstruction fails proportionally to the deviation Δ.
- Without PE:
  (8) $$\begin{array}{cc} {\mathrm{Error}}_{\mathrm{noPE}} & ={∥f_{\mathrm{noPE}}\left(I^{\prime }\left(x_{0},y_{0}\right);\theta \right)-I^{\prime }\left(x_{0},y_{0}\right)∥}^{2}\\ \; & ={∥\mu_{\mathrm{global}}-\left(\mu_{x_{0},y_{0}}+\Delta \right)∥}^{2} \end{array}$$
  Since the model lacks spatial information, it learns to reconstruct based on a global average $\mu_{\mathrm{global}}$, which aggregates feature statistics across the entire training set. If $\mu_{x_{0},y_{0}}+\Delta$ resembles values seen elsewhere in the training data, the model may still reconstruct it with low error—failing to recognize it as anomalous. As a result, the reconstruction error becomes inconsistent and depends on how much the local anomaly deviates from the global distribution.

Thus, only the PE-enabled model is able to assign a high reconstruction error to spatially inconsistent input, enabling more reliable anomaly detection at test time.

### 3.4. Autoencoder Architecture

The proposed model is a convolutional autoencoder designed to reconstruct fluoroscopic images while capturing their underlying structural patterns. It adopts an encoder–decoder architecture, where the encoder extracts a compressed representation of the input, and the decoder reconstructs the original image. The reconstruction quality serves as an indicator of structural deviation, enabling the identification of unseen elements such as bolus or residue that differ from the learned background.

The encoder consists of three convolutional blocks: the first applies a 2D convolution with 64 filters of size 3×3, followed by batch normalization, ReLU activation, and a dropout layer (rate = 0.1). Subsequent layers use 128 and 256 filters (kernel size 3×3) with batch normalization and ReLU activation. Each block is followed by 2×2 max-pooling to reduce spatial resolution and promote hierarchical feature abstraction.

The decoder mirrors this design, employing transpose convolutions for upsampling. Skip connections are included between encoder and decoder layers at matching resolutions, ensuring that fine-grained spatial details are preserved during reconstruction. Each decoded feature map undergoes batch normalization and ReLU activation to enhance stability and nonlinearity. Finally, a 3×3 convolution with 3 output channels and sigmoid activation generates the reconstructed frame.

This architecture balances efficiency and accuracy: max-pooling layers capture high-level abstractions, skip connections mitigate information loss, and dropout enhances generalization. Together, these design choices ensure that reconstructed boundaries remain sharp and clinically relevant rather than overly smoothed.

### 3.5. Training Strategy

To effectively train the model, we use a dataset of 10,000 fluoroscopic frames. All selected frames exclude the bolus, enabling the model to learn the natural background structure of the swallowing anatomy. To ensure the model captures an accurate representation of the background while preserving critical anatomical details, we define a hybrid loss function that combines two components:

1. Mean squared error (MSE):
  (9) $$L_{MSE}=\frac{1}{N}\sum_{i=1}^{N}{\left(y_{i}-{\hat{y}}_{i}\right)}^{2}$$
  This term promotes pixel-wise similarity between the input and its reconstruction, ensuring general fidelity.
2. Edge-Aware Loss:
  (10) $$L_{Edge}=\sum_{i}\left|\nabla y_{i}-\nabla {\hat{y}}_{i}\right|$$
  where ∇ denotes the image gradient. This term encourages preservation of sharp anatomical boundaries and reduces excessive blurring in the reconstructed images.
  The final loss function is a weighted sum of these components:
  (11) $$L_{Total}=L_{MSE}+\lambda L_{Edge}$$
  where λ is a weighting factor that balances edge preservation. In our experiments, we empirically set λ=0.05 based on validation set performance to achieve desired reconstruction quality.

To promote generalization, training is conducted on fluoroscopic frames collected from a diverse cohort of participants, allowing the model to capture anatomical and imaging variability. To further improve robustness, we apply light Gaussian noise augmentation during training. This encourages reliance on structural and spatial cues rather than memorization of fine-grained intensity patterns. By perturbing local pixel details without altering global structure, this augmentation discourages over-reliance on specific image regions and fosters a more comprehensive understanding of background characteristics.

Model training was performed using the Adam optimizer with an initial learning rate of 0.01. This relatively large starting value was chosen empirically to enable rapid convergence in the early training stages, given the small size of the dataset and the lightweight nature of the network. To prevent instability, a learning rate scheduler (ReduceLROnPlateau) was applied: if the validation loss did not improve for 10 consecutive epochs, the learning rate was automatically reduced by a factor of 0.5. This adaptive adjustment allowed fine-grained updates in later epochs, ensuring continued convergence without premature stagnation. Training was conducted with a batch size of 16 for 100 epochs. No weight decay was applied, and the Adam momentum parameters were left at their default values ($\beta_{1}=0.9$, $\beta_{2}=0.999$). Early stopping was not enforced; instead, the final model was selected based on the lowest loss observed during training.

Once trained, the model learns to reconstruct only the background features encountered during training. At inference, the presence of unseen structures—such as the bolus or residue—produces reconstruction errors, which are leveraged for segmentation. These deviations are quantified using a pixel-wise error map, computed as the absolute difference between the input frame and its reconstruction. To highlight anomalous regions, an adaptive thresholding strategy is applied, wherein the threshold is dynamically determined based on the distribution of reconstruction errors. In practice, we validated several thresholding strategies and found that using the mean reconstruction error, identified through a 10% validation subset of the test data, provided the most consistent performance. This yields a binary segmentation map in which high-error regions—corresponding to unrecognized structures—are emphasized while well-reconstructed areas are suppressed. Finally, a region-of-interest (ROI) mask is applied to restrict analysis to anatomically relevant areas, defined based on anatomical landmarks and guided by speech-language pathology expertise in swallowing physiology. Specifically, the left, right, and upper borders of each frame were excluded, as VFSS protocols introduce gaps between these borders and the participant’s head and neck.

### 3.6. Model Evaluation

To evaluate the performance of the proposed framework, we used a dataset comprising 305 VFSS frames, including 250 frames containing bolus and 55 frames containing bolus residue, collected from individuals representing both healthy and dysphagic subjects. The model detects both bolus and residue using the same reconstruction-based mechanism, without requiring architectural modifications or task-specific tuning. Ground truth segmentation masks, validated by certified speech-language pathologists, were compared against the binary segmentation outputs produced by the model. Performance was quantitatively assessed using several evaluation metrics, including accuracy, sensitivity, specificity, IoU, and DSC:

- **Accuracy** measures the overall correctness of the segmentation by computing the proportion of correctly classified pixels:
  (12) $$Accuracy=\frac{TP+TN}{TP+TN+FP+FN}$$
- **Sensitivity** quantifies the model’s ability to correctly identify bolus regions:
  (13) $$Sensitivity=\frac{TP}{TP+FN}$$
- **Specificity** evaluates how well the model distinguishes non-bolus regions from the background:
  (14) $$Specificity=\frac{TN}{TN+FP}$$
- **IoU** measures the overlap between the predicted bolus region and the ground truth:
  (15) $$IoU=\frac{TP}{TP+FP+FN}$$
- **DSC** provides a balanced measure of segmentation performance by emphasizing both precision and recall:
  (16) $$DSC=\frac{2TP}{2TP+FP+FN}$$

While accuracy offers a general measure of correctness, it can be misleading in the presence of class imbalance, particularly due to the predominance of background (black) pixels in the segmentation masks. In such cases, IoU and DSC provide more informative metrics, as they offer a balanced evaluation of both foreground and background regions.

In addition to segmentation accuracy, we also assess the computational efficiency of the proposed framework. A lightweight model with a reduced parameter count and lower computational complexity is desirable for clinical settings, enabling fast and cost-effective inference without reliance on high-performance hardware. To evaluate the feasibility of real-time deployment, we report key efficiency metrics, including the number of trainable parameters, floating point operations (FLOPs), and average inference time.

## 4. Results

### 4.1. Bolus and Bolus Residue Segmentation

Table 1 summarizes the segmentation performance for both bolus and bolus residue, as well as the computational efficiency of the proposed model. While residue segmentation achieves high accuracy, sensitivity, and specificity, the IoU and DSC scores are relatively lower. This reduction is primarily attributable to the very small size of residue regions within each frame; even minor discrepancies between the predicted and ground truth masks can lead to a substantial drop in overlap-based metrics. Due to the smaller size of the residue dataset, we further assessed robustness by applying bootstrapping (1000 resamples with replacement) on the residue test set, which confirmed that the IoU (≈50.3%) and DSC (≈66.6%) remained consistent with the originally reported results.

**Figure 4:**
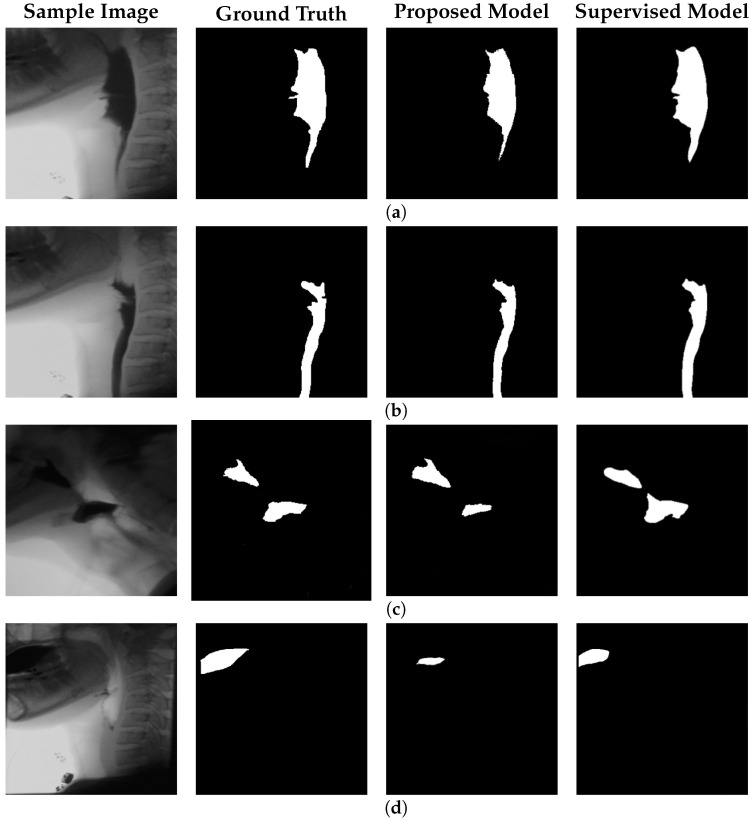
Qualitative results of bolus segmentation using the proposed model with positional encoding. Each group of four columns corresponds to a test case. From left to right within each group: (**a**) original VFSS frame, (**b**) ground truth segmentation mask, (**c**) prediction by the proposed unsupervised model, and (**d**) prediction by the supervised model from [14], representing a state-of-the-art benchmark.

Figure 4 illustrates representative test frames containing bolus, along with their corresponding ground truth annotations, predictions from the proposed model, and segmentation results from the supervised model presented in [14], which reports state-of-the-art performance for automatic bolus segmentation. Notably, the supervised model was originally trained on a dataset acquired using the same VFSS imaging device and curated by the same clinical team, making this comparison particularly relevant and reliable. This consistency ensures that observed differences in performance are attributable to methodological differences rather than domain shift or annotation bias.

Figure 4c illustrates a case of bolus discontinuity, where two distinct bolus regions appear within the same frame. This spatial separation increases the complexity of the detection task compared to single-region bolus cases. Despite this challenge, the model demonstrates reasonable accuracy in identifying both regions. Overall, the model performs well in bolus detection; however, its accuracy declines in frames where the bolus remains within the oral cavity, as shown in Figure 4d. This limitation is primarily due to the lower spatial resolution of the imaging equipment in the oral region, which reduces the visibility of fine structural details and contrast variations necessary for accurate segmentation. Notably, the supervised baseline model also exhibits reduced performance in this scenario, highlighting the inherent difficulty of detecting the bolus in the oral phase. As a result, both models struggle to reconstruct and segment the bolus accurately in this region.

**Figure 5:**
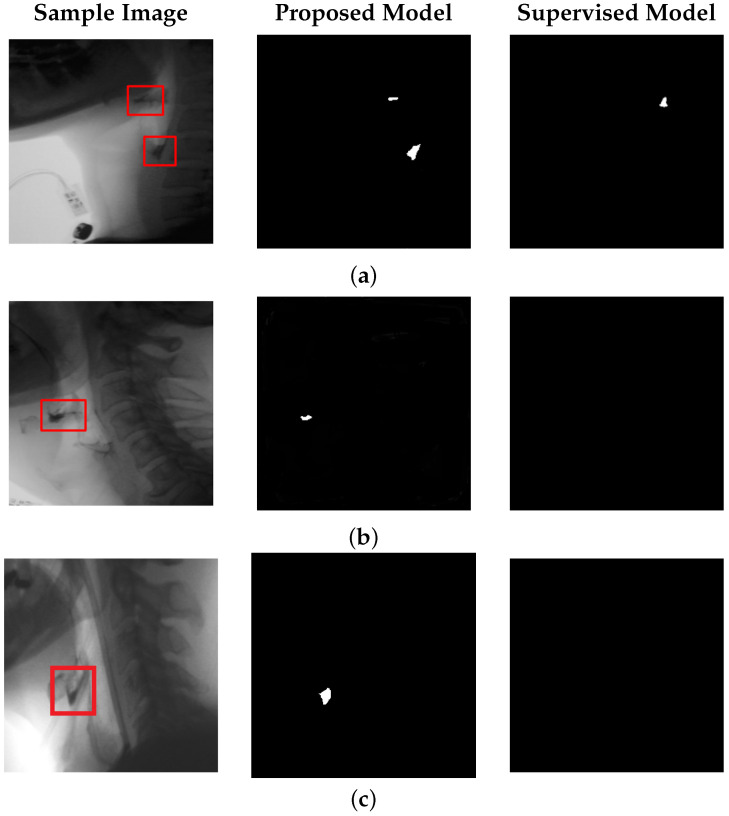
Performance of the proposed model with positional encoding for bolus residue segmentation. Each row shows a sample test frame, the corresponding residue segmentation produced by our model, and the output from the supervised model [14]. (**a**) illustrates a case with discontinuous bolus residue, while (**b**,**c**) depict frames containing a single residue region.

Figure 5 focuses on bolus residue segmentation, showcasing output masks generated by our unsupervised model. The last column also includes results from the supervised model [14] applied to the residue detection task. Although supervised models perform well in bolus tracking, they often struggle with residue detection. These models are typically trained on frames containing well-defined bolus structures, making them less effective at identifying residue, which tends to be smaller, lower in contrast, and more spatially dispersed. As a result, supervised models often fail to generalize, overfitting to the visual characteristics of the bolus and missing the subtle, irregular appearance of residue regions.

In contrast, our unsupervised approach does not rely on explicit object annotations. It learns inherent structural patterns from the data itself, without being constrained by predefined labels. This allows the model to remain sensitive to anomalies—enabling detection of both bolus and bolus residue—while maintaining robustness to normal anatomical variability observed during the swallowing process. Statistical testing confirms that, for residue detection, our method significantly outperforms the state-of-the-art supervised baseline in IoU and DSC metrics (p<0.05, paired *t*-test; results for the supervised model are detailed in the next subsection).

### 4.2. Comparison with Fully Supervised Approaches

To compare our framework with a fully supervised approach, we used the model introduced in [14], which reports state-of-the-art performance in bolus segmentation. Access to the pretrained weights of the U-Net architecture with a MobileNetV2 encoder–decoder was provided, allowing for direct evaluation on our test set. The average segmentation results obtained using this model are reported in Table 2. Additionally, computational efficiency metrics were adopted from the original study to enable a clearer comparison with the proposed model. All experiments were conducted on an Nvidia GeForce RTX 3080 Ti GPU for both supervised and unsupervised methods.

## 5. Ablation Study

### 5.1. Unsupervised Segmentation Without Global Context Awareness

To assess the contribution of global context awareness, we conducted an ablation study by removing the positional encoding channel from the input while keeping the rest of the architecture unchanged. In this variant, the model was limited to local feature extraction through convolutional layers. As shown in Table 3, this modification led to a decline in segmentation performance, particularly in IoU and DSC metrics.

The primary reason for this performance decline is that, without global context awareness, the model relies on globally aggregated feature statistics rather than spatially localized expectations. Consequently, it struggles to distinguish the bolus from surrounding tissues with similar color or texture. As we demonstrated earlier, this can lead to partial bolus reconstruction in some cases, depending on its similarity to globally familiar patterns. This inconsistency reduces the contrast in the error map between bolus and non-bolus regions, making segmentation less effective than the context-aware version.

Our approach to global information capturing employs positional encoding as an efficient, lightweight alternative to computationally intensive global feature extraction techniques. Unlike methods that depend on high-capacity architectures such as self-attention mechanisms, our strategy introduces global spatial context without adding learnable parameters or increasing computational overhead. Since positional encoding is derived directly from the spatial dimensions of the input image, it eliminates the need for costly feature aggregation. By simply concatenating these precomputed features as additional input channels, the model retains its original structure while gaining spatial awareness.

### 5.2. Sensitivity to Geometric Augmentations

While data augmentation is widely used to enhance model robustness, our framework exhibits sensitivity to geometric transformations due to its reliance on fixed positional encoding. Augmentations such as flipping or rotation modify the spatial layout of the input without updating the corresponding positional encoding, resulting in inconsistencies between visual features and their encoded locations.

**Figure 6:**
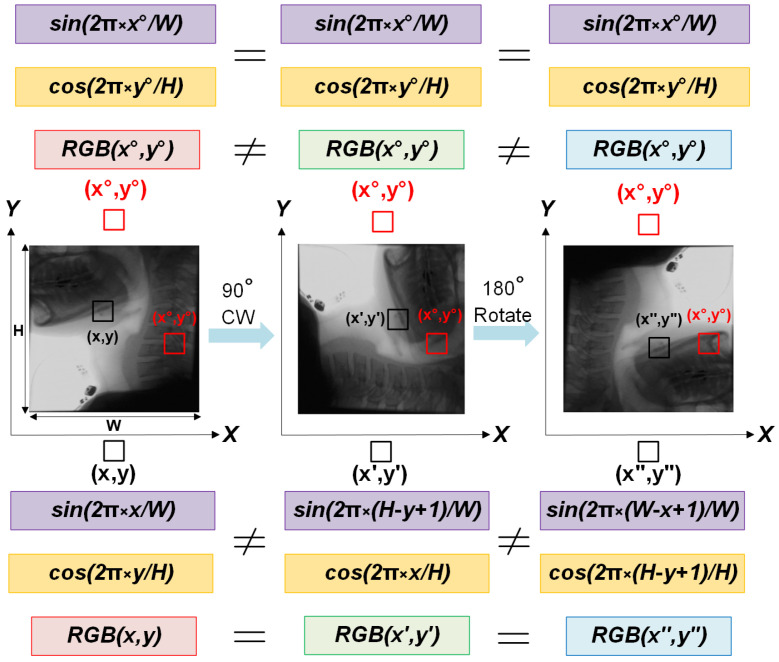
Visualization of how geometric augmentations (here, flipping) introduce inconsistencies between visual features and positional encoding. The same anatomical region may be associated with different positional cues, and conversely, the same positional coordinates may correspond to different anatomical content. This mismatch can confuse the model during training and contribute to segmentation failures at inference time. We use “RGB” notation here to represent pixel intensity values for visualization purposes only.

Figure 6 illustrates how flipping alters the spatial relationship between image content and positional encoding. This misalignment introduces inconsistencies that can confuse the model during training. At inference time, such discrepancies led to bolus detection failures in our experiments.

## 6. Discussion

This study demonstrates that segmentation of both bolus and bolus residue in VFSS recordings can be effectively achieved through an unsupervised learning approach, without relying on pixel-level annotations. By leveraging anomaly detection via a CNN-based autoencoder trained exclusively on non-bolus frames, the model learns to reconstruct normative swallowing anatomy while treating unobserved structures, such as the bolus, as spatial anomalies during inference. This enables effective localization using reconstruction error maps, eliminating the need for manual mask annotations while maintaining strong segmentation performance.

A notable strength of this framework is its clinical scalability: it was trained using diverse frames drawn from participants with varying swallowing profiles, and required no segmentation labels—making it easier to extend to larger or more heterogeneous datasets. However, a key challenge in this type of anomaly detection is ensuring that typical anatomical movements, such as hyoid excursion or epiglottic inversion, are not falsely flagged as anomalies. To mitigate this risk, we incorporated a diverse training distribution and applied an ROI mask during postprocessing to constrain the model’s focus to the pharyngeal area, thereby reducing false positives caused by background artifacts or unrelated motion.

To further enhance spatial sensitivity, positional encoding was incorporated as an additional input channel. This mechanism provides a consistent spatial reference for the model, complementing the local feature extraction capabilities of the CNN architecture. While this addition improves detection fidelity by anchoring features to specific regions, it introduces sensitivity to geometric transformations, such as rotation or flipping. However, because the training data was sufficiently diverse, we observed that moderate variations—such as changes in head tilt, or swallowing posture-did not adversely affect performance.

In practice, the potential limitations introduced by positional encoding can be analyzed in two scenarios. In the first case, the trained model is applied to data collected within the same medical center or any center with similar imaging protocols, field of view, and patient positioning. Here, the positional encoding serves as a reliable spatial guide, and the model can generalize well to new patients in all these medical centers. It is worth noting that anatomical framing and acquisition protocols are generally similar across centers, though not always identical. In the second scenario, the model’s generalizability will be affected if deployment occurs in a center with significantly different imaging setups or acquisition protocols. Nevertheless, the same unsupervised training procedure can be repeated locally to adapt the model. Since our method does not rely on manual annotations and employs a lightweight architecture, retraining is both efficient and practical. Therefore, while positional encoding may initially appear to limit generalizability, it can in fact be easily recalibrated, enabling the approach to adapt flexibly across diverse clinical environments.

Our proposed model achieves performance in bolus segmentation that is comparable to a well-established supervised approach, but it drastically outperforms the supervised method in bolus residue detection. This improved residue detection suggests that learning from negative space—rather than relying on sparse or potentially biased residue annotations—may provide a more generalizable and robust pathway for detecting underrepresented clinical phenomena. This insight highlights the potential of label-free, anomaly-based frameworks for detecting subtle diagnostic markers in VFSS and may extend to other medical imaging domains where annotated data are limited or unreliable.

One notable advantage of the proposed model is its computational efficiency. With only 1.71 million parameters and 0.0034 GFLOPs, it is substantially smaller and faster than conventional supervised models. Its short inference time supports real-time deployment, making it suitable for integration into existing imaging systems, including portable platforms and clinical infrastructure. This efficiency reduces computational overhead, streamlines workflow, and improves patient throughput—key advantages in high-demand settings such as radiology and swallowing disorder clinics.

Several avenues exist for future research. The current segmentation output could be extended to derive additional clinically relevant metrics, such as bolus transition duration and speed, which may assist in detecting abnormal transit patterns indicative of neuromuscular disorders, esophageal motility issues, or neurodegenerative conditions. Future work could also address aspiration events, including segmentation of aspirated bolus and estimation of its volume to support risk assessment and severity grading. Beyond bolus segmentation, this anomaly-based framework can potentially be generalized to other medical image segmentation tasks by redefining the target structure as an anomaly relative to normative anatomy. By integrating unsupervised learning principles, spatial encoding, and domain-specific ROI constraints, this approach offers a promising direction for achieving reliable segmentation performance without the need for extensive manual annotation. With appropriate modifications, the core methodology may also be adapted to non-medical applications, broadening its potential impact.

## 7. Conclusions

In this study, we proposed a machine learning framework for bolus segmentation and residue detection in VFSS recordings that does not require pixel-wise annotation. To our knowledge, this is the first study to successfully detect bolus residue alongside bolus segmentation, addressing a significant gap in the literature while also achieving notable detection performance. Our method achieves bolus segmentation results comparable to fully supervised approaches while significantly reducing the need for manual labeling. Without any architectural modifications, the same framework enables robust detection of bolus residue, outperforming well-established supervised models and offering actionable feedback for clinical decision-making in dysphagia assessment. Unlike supervised models, which often overfit to high-contrast bolus regions seen during training, our method learns from negative space—regions typically devoid of bolus—resulting in a more generalized and nuanced understanding of image context. This enables better identification of residue, which is usually small, faint, and spatially scattered. By not relying on direct residue labels, the model avoids biases inherent in sparsely annotated datasets and can detect sparsely represented features with greater robustness. Additionally, computational analysis shows that the model is lightweight and achieves 1.5× faster inference, reinforcing its suitability for real-time clinical deployment. Future work will focus on scaling the dataset, incorporating additional physiological indicators, and optimizing the framework for clinical integration.

## Figures and Tables

**Table 1:** Test time segmentation performance and computational efficiency of the proposed unsupervised model with global context awareness. The 95% confidence interval is reported for each metric.

Bolus Segmentation Performance	
Accuracy (%)	98.08 (97.12, 99.01)
Sensitivity (%)	66.55 (64.42, 68.27)
Specificity (%)	99.48 (98.36, 99.65)
IoU (%)	61.49 (60.01, 63.45)
DSC (%)	72.31 (70.26, 74.18)
Residue Segmentation Performance	
Accuracy (%)	99.88 (99.36, 99.98)
Sensitivity (%)	90.37 (88.84, 91.92)
Specificity (%)	99.90 (99.41, 100.00)
IoU (%)	51.89 (50.37, 53.44)
DSC (%)	66.33 (64.80, 67.87)
Computational Efficiency	
Parameters (M)	1.71
FLOPs (G)	0.0034
Inference Time (ms)	5.30

**Table 2:** Test time segmentation performance and computational efficiency of the fully supervised model from [14]. The 95% confidence interval is reported for each metric.

Bolus Segmentation Performance	
Accuracy (%)	98.55 (97.54, 99.41)
Sensitivity (%)	76.52 (74.12, 79.01)
Specificity (%)	99.28 (98.31, 99.95)
IoU (%)	66.35 (64.10, 68.84)
DSC (%)	76.18 (73.72, 78.59)
Residue Segmentation Performance	
Accuracy (%)	99.74 (98.43, 100.00)
Sensitivity (%)	12.02 (9.43, 14.52)
Specificity (%)	99.98 (98.81, 100.00)
IoU (%)	11.28 (8.88, 13.75)
DSC (%)	20.28 (17.79, 22.69)
Computational Efficiency	
Parameters (M)	27.17
FLOPs (G)	6.63
Inference Time (ms)	7.83

**Table 3:** Test time segmentation performance and computational efficiency of the unsupervised model without global context awareness. The 95% confidence interval is reported for each metric.

Bolus Segmentation Performance	
Accuracy (%)	95.63 (93.13, 98.05)
Sensitivity (%)	52.87 (49.37, 56.37)
Specificity (%)	98.84 (96.34, 99.96)
IoU (%)	49.58 (46.08, 53.08)
DSC (%)	61.89 (58.39, 65.39)
Residue Segmentation Performance	
Accuracy (%)	99.80 (98.76, 100.00)
Sensitivity (%)	56.01 (51.42, 60.38)
Specificity (%)	99.92 (98.79, 100.00)
IoU (%)	22.31 (17.77, 26.66)
DSC (%)	35.14 (30.53, 39.63)
Computational Efficiency	
Parameters (M)	1.71
FLOPs (G)	0.0034
Inference Time (ms)	5.28

## Data Availability

The raw data supporting the conclusions of this article will be made available by the authors on request.

## Abbreviations

The following abbreviations are used in this manuscript:

VFSS	Videofluoroscopic Swallowing Study
IoU	Intersection over Union
DSC	Dice Similarity Coefficient
CNN	Convolutional Neural Network
